# Exploring the system capacity to meet occupational health and safety needs: the case of the ready-made garment industry in Bangladesh

**DOI:** 10.1186/s12913-019-4291-y

**Published:** 2019-06-28

**Authors:** Sadika Akhter, Shannon Rutherford, Cordia Chu

**Affiliations:** 1 School of Science and Environment, Griffith University, Brisbane, Australia; 2 International Centre for Diarrhoeal Disease Research, Bangladesh, 68 Shaheed Tajuddin Ahmed Sarani, Dhaka, 1212 Bangladesh; 3 School of Medicine, Griffith University, Brisbane, Australia

## Abstract

**Background:**

Since the 2013 Rana Plaza incident in Bangladesh, the government of Bangladesh has been under pressure to improve health and safety conditions for workers in the ready-made garment industry. Its efforts have focused heavily on structural safety of the buildings but have largely ignored broader occupational health system issues. This qualitative study explores contextual factors and system challenges that create barriers for ensuring a healthy and safe workplace in the ready-made garment industry in Bangladesh.

**Methods:**

Data were collected through key informant interviews (*n* = 14) with government officials from the Department of Inspection for Factories and Establishments (DIFE), factory employers, factory doctors and representatives from the Bangladesh Garment Manufacturers and Exporters Association (BGMEA). A thematic analysis was conducted using Atlas-ti v 5.2.

**Results:**

A thematic analysis suggests that the capacity of the DIFE to provide adequate occupational health services remains a problem. There is a shortage of both appropriately trained staff and equipment to monitor occupational health and safety in factories and to gather useful data for evidence-based decision-making. Another barrier to effective occupational health and safety of workers is the lack of cooperation by employers in recording data on workers’ health and safety problems. Finally, government officials have limited resources and power to enforce compliance with regulations. Such deficiencies threaten the health and safety of this important, largely female, working population.

**Conclusion:**

This case example focused on the valuable ready-made garment industry sector of Bangladesh’s economy. It identifies the critical need for occupational health system strengthening. Specifically system capacity needs to be improved by both increasing human resources for in-factory hazards and health monitoring, regulatory inspection, enforcement, and improved training of government officials around monitoring and reporting.

**Electronic supplementary material:**

The online version of this article (10.1186/s12913-019-4291-y) contains supplementary material, which is available to authorized users.

## Background

According to the International Labour Organization (ILO), globally every year over 2.3 million people suffer from occupational accidents and work-related diseases, thousands of people die every day due to occupational accidents, and about 4% of the world’s gross domestic product (GDP) is lost in direct and indirect costs of occupational accidents and work-related diseases [1]. In developed countries, data related to workplace health and injury is readily available [2, 3]. However, resource limited countries face significant challenges in collecting reliable data on occupational health and safety issues [2, 4].

Bangladesh is moving towards an industrialized economy and occupational health problems have emerged as a concern in some of its industries [5]. Among these industries the biggest is the ready-made garment (RMG) industry, earning USD 30.16 billion in 2017–18 and accounting for over 83% of the nation’s export earnings [6] . It employs around 4 million workers, 80% of whom are women [7]. Despite the phenomenal growth of the RMG sector, it is characterized by fire incidents and poorly constructed unsafe workplaces [8].

Recent industrial incidents such as the Rana Plaza collapse in 2013, which killed over 1000 workers and injured many thousands more, highlight the problems the industry faces. This incident came at a critical time, when the government was trying to reassure global buyers by ensuring safe and acceptable working conditions in the RMG sector [9].

Consequently, the government of Bangladesh has worked to enhance the capacity of the Department of Inspections of Factories and Establishments (DIFE) under the Ministry of Labour and Employment [10]. These initiatives include: upgrading the inspectorate to a department in 2014, creating new positions of inspectors and increasing the budget, recruiting almost two hundred new inspectors including female inspectors, and developing an inspection check list for an information management system, and providing more essential equipment such as motorcycles to facilitate factory inspections [11]. These are positive steps toward improving the OHS system in Bangladesh. The main objective of this study is to understand the OHS system related d challenges that face the industry and government in managing the occupational health and safety services in the country based on the WHO global plan of action framework for workers’ health.

## Methods

### The conceptual framework

In 1994, the World Health Organization (WHO) proposed a global strategy on occupational health for all to meet ten objectives [12]. These are: developing international and national policy; creating a healthy working environment, healthy work practices and promotions; strengthening occupational and health and safety (OHS) services; developing support services for OHS, developing OHS standards based on risk assessment; development of human resources; establishment of registration and data systems; development of information services of data and raising awareness about OHS through sharing information; conducting research and finally developing collaboration [12]. To understand the OHS system status and challenges in the RMG sector in Bangladesh, the present study explores three elements of the framework: i) occupational health and safety services and capabilities ii) surveillance and the national reporting system iii) collaboration between different stakeholders and competing realities. It does so by analyzing data collected through key informant interviews from diverse stakeholders.

### Study design and data collection

A qualitative research approach was used in order to identify key issues relating to the OHS system capacity as it relates to the ready-made garment industry in Bangladesh [13, 14]. Data collection was conducted from December 2015 to June 2016 in Dhaka district, Bangladesh. The principal researcher (SA) collected data with a university graduate with experience in conducting qualitative interviews and trained for data collection. During the training the purpose of the study and qualitative interviewing techniques were described. Role plays were done during the training session to train the data collector to understand how to conduct an interview and to familiarize her with the interview guide-line. Open-ended guidelines were developed (Additional file 1) for the interviews. The major topics of the interview guideline were: a) the current situation of OHS system readiness to provide services b) challenges they face in developing an effective OHS system, and c) current practices of data collection and the national reporting system. A pre-test of the interview guideline was also conducted before the actual data collection. The interviews were conducted face-to-face at the workplace of study participants according to their preference and additional probing questions were asked when a new topic emerged during the interviews. Each interview lasted around one and half hours. The interviews were recorded with permission from each of the study participants.

### Key informant interviews

Fourteen key informant interviews were conducted among government and industry officials who are involved in the ready-made garment industry and/or managing occupational health and safety (OHS) in Dhaka district. They included: three government officials from the Department of Inspections of Factories and Establishments (DIFE) under the Ministry of Labour and Employment, four industry employers, four factory doctors and three representatives from the Bangladesh Garment Manufacturers and Exporters Association (BGMEA).

### Sampling

These key informant interviews were sought to elicit a range of expert opinions about OHS system related challenges for this sector. As such, informants were selected using a purposive sampling method because of their potential ability to provide detailed insight and first-hand experience in OHS system and program implementation in this sector in Bangladesh. In contrast, the logic for purposeful sampling for this research was selecting information-rich participants to explore in depth [15]. The guidelines to determine sample sizes for qualitative research are not as rigorously codified as sample sizes are for hypothesis testing research, where power calculations based on a priori knowledge of distribution and variability of key dependent variables is essential. Usually the qualitative research focuses in-depth on relatively small samples, even it could be single cases (*n* = 1) [16]. Instead the concept of data saturation was deemed appropriate – data collection was halted when no new or additional information was found from the key informant interviews [17].

### Data analysis

Data collection, organization and analysis were done concurrently. All interviews were transcribed verbatim in Bengali to prepare transcripts and text was translated by the researcher into English. Detailed notes were also taken during the interviews. Thematic analysis was conducted to analyse data [18]. All transcripts were read and re-read to develop a code list from the textual data [19]. The code list was developed by two independent researchers to identify emerging themes and to ensure interpretation of data. The two code lists then were reviewed and compared with each other together to identify common themes to reach fair interpretation of the data. The main themes and sub-themes of data are discussed below along with relevant quotes. The simultaneous process of data collection, transcriptions, identifying codes and themes enabled a thorough data analysis [20].

## Results

### Occupational health and safety services and capabilities

#### Insufficient human resources

Informants described the establishment of DIFE in the 1970s under the Ministry of Labour and Employment. It was known as the Labour Department. At that time it was divided into three departments: Labour, Inspection for Factories and Establishments and Registration of Trade Unions. The Inspections division began to work separately as a directorate in the same year while the other two divisions were combined. The Directorate of Inspections was headed by a Chief Inspector of Factories and Establishments and a Deputy Chief Inspector under the Chief Inspector. The directorate covered the entire country with a total of 250–300 government officials until 2014. The department increased the number of staff following the industrial incident of Rana Plaza in 2013.

Despite considerable progress since 2014, there remains a shortage of qualified staff, especially doctors in DIFE. According to the key informants from the government department five doctors should be posted in the Dhaka district office to monitor the health and safety of workers but at the time of interview no doctors were actually working in this office. A key reason provided by informants is that doctors perceive there are no career prospects in DIFE and once posted to the DIFE they lobby to instead work with the Ministry of Health and Family Welfare, where there is a better career path. A consequence of this lack of medical knowledge in the DIFE was identified by one informant:

*“The health related issues and problems need to be reported by the doctors. There is no doctor currently working with the DIFE in Dhaka district office. We are currently collecting data about the health, injury and safety related issues with the help of the factory inspectors. These inspectors are from academic background of science like zoology, chemistry and botany. They do not understand deeply the issues of health and safety of the workers*.”

There is also a shortage of suitably qualified engineers. Following the Rana Plaza incident more emphasis was given to building safety. However, the Dhaka district office does not have an appropriately qualified number of engineering staff to assess structural safety of buildings. One informant said,

“*Most of the engineers who are working with the safety section have an educational background of electrical or chemical engineering. The building structure issues will be better understood by a civil engineer. But we do not have any engineer with civil engineering background, which is a big challenge. We need ten engineers but we have five engineers.”*

#### Training and skill deficits

Key informant interviews revealed that the level of training and skills of key staff at the Dhaka district office regarding OHS, Monitoring and Evaluation (M&E), and data management varies substantially. There are few courses on OHS, labour law, or M&E programs at tertiary education institutions in Bangladesh. Training related to OHS is generally obtained by staff through workshops and online short courses. Some of the staff at the national and district level have received basic training in OHS through an online training course, but their analytical skills are reported to be very weak. The government officials informed that more training is needed at the individual level on collecting, analysing and interpreting data. The following responses from a government official demonstrate this need:

*“Our training is not adequate. We need more training on OHS. As we are moving towards an industrialised country slowly, the occupational health problem will be a big challenge for us in the future. We need skilled and trained staff for OHS. Currently we are receiving six-month distance courses with the help of ILO on OHS. The ILO also provides a two-month course on labour-inspection. It is helping to improve our skills.”*

#### Shortage of equipment

The government officials reported that a shortage of equipment at all levels limits the capacity to assess the health and safety related problems of the RMG industry. Until motorbikes were recently provided, even visiting the widely scattered factories was a big problem. Informants indicated that DIFE staff has laptops, computers and Wi-Fi connection facilities in their offices but specific OHS monitoring equipment was absent or severely limited. Study participants reported that the Dhaka office has only seven teams to monitor OHS issues at the RMG factory level and though they need approximately seven sets of monitoring and safety equipment such as sound meters, light meters and pairs of safety boots, they only have five of each. The informants presented their views on how they manage it:

*“We still have a shortage of other equipment such as light meters, sound meters, helmets, and boots. We do not have enough equipment for all staff. We use them by rotation, which is a problem in ensuring a quality visit to the factory.”*

### Surveillance and the national reporting system

#### Factory monitoring tool

Key informant interviews revealed an awareness that data is necessary for effective policy development, but that there is little reliable data available for OHS in Bangladesh. A number of informants reported that before the Rana Plaza incident there was no standardized factory monitoring tool to collect data on occupational health problems of garment workers. In the absence of such a tool, the health inspector commonly collected data in an adhoc way in notebooks. After the Rana Plaza incident and under pressure from overseas donors, the DIFE developed a factory monitoring checklist (a hard copy form with over 100 questions, with completed forms entered into a computer following data collection) which is now used by factory inspectors. However, most of the informants believed that its emphasis is on building structural safety issues with limited data collection on other health and safety issues relevant to this sector. Further, coverage and frequency of data collection was highlighted as an important issue:

*“We developed a checklist with the help of our development partners. The checklists have 125 questions. We collect data on the building structure, recruitment, child labour, and the maternity benefits. We do not collect enough data* [i.e. the checklist does not include] *on occupational health and safety related disease. As we have a serious shortage of manpower, our inspector cannot visit the factories regularly to collect data. It takes two to three days to complete the checklist.”*

#### Data collection and reporting

All informants talked about the lack of data on health and safety problems of the workers. There are no within-factory standard procedures for collecting, storing, and reporting data on incidents, specific OHS hazards or health issues. In their own words, informants explained their views on the issue this way:

*“You’ll find that the factory register book is blank. The factories do not report to us about the health and safety problems of their workers.”*

The inspecting body, DIFE, does not collect this data either, due to insufficient staffing despite the recent workforce expansions. Informants reported potential health problems based on their own experiences, but without comprehensive data it is difficult to manage hazards and associated health impacts and diseases faced by the workers at individual factories. As indicated by one factory inspector:

*“I visited a garment factory many times. I had difficulty in breathing due to inhaling fabric dust. We need reliable data but we do not have data on the health problems of the workers in our hand now.”*

Further to the absence of individual factory level information for local hazard and health risk management, informants also identified the absence of a national system for reporting, assessing and sharing the data in order to make policy, design strategy and interventions to address the health problems of the workers.

### Perspectives of different stakeholders and competing realities

#### Government officials

Government officials from the DIFE reported concerns about implementing laws. Under the current regulation system they feel powerless to enforce their own regulations. The DIFE does not have direct power to take any action against any factory in disputes about labour rights or protecting the health and safety of workers. One government official indicated:

“*If we come to know about the health problems of the workers, we request the owner to pay the treatment cost. We request them to pay the maternity benefit to pregnant workers. If they do not listen to us, we can only file a case in the labour court. We do not have enough administrative power to punish the owner instantly.”*

Informants mentioned that factory managers readily accept referral to court, knowing that the court is overloaded and it will take a long time, if ever, for the matter to be resolved. Even if the business owner is found guilty, the highest penalty is only 25,000 BDT (USD 312). This is not an effective incentive to comply with the law for such large and profitable businesses.

#### Factory managers

Factory managers appeared reluctant to keep adequate records about workers’ health and safety. They indicated that there are doctors in their factories to treat the workers when they are sick and further probing revealed that they are concerned that if they keep records, the government would insist that they compensate workers for their health problems. This would have consequences for their profits as explained by an employer:

*“If the price of a shirt is 100 taka, we spend 13 taka for the labour cost; the rest of the cost is for accessories. We import all the accessories from other countries. If we could produce the accessories locally we could spend more money on the workers’ health and safety issues. We try to make a profit from this 13 taka because the rest of the costs are fixed.”*

Thus it appears that factory managers are not motivated to record injuries or illness as they are concerned about future compensation payouts and related impacts on production costs.

#### Factory doctors

The factory doctors are aware of worker health issues and the need for information in order to provide health services for the RMG workers. However they feel powerless to do anything about the situation as they are directly employed by the factory owner. The doctors have not been asked by their factory management to collect or forward data about the health problems of the workers that they see, even though the factory is required to report such data. All doctors expressed their concerns about health issues for the workers with one specifically expressing concern about the long term health consequences of working in the industry:

*“Most of the women leave the factory job after the age of 35. They cannot continue their work in this sector after this age because of the hard work and the working environment. They constantly inhale cloth dust. You will hardly find a woman who is forty years old or above working in this sector. The government needs to develop a strategy to address the health problems of the workers. Otherwise, this population will be a huge burden of disease for the country in the future.”*

#### Bangladesh garment manufacturers and exporters association (BGMEA)

Informants from the BGMEA indicated that according to the law, OHS issues must be addressed collaboratively by government officials, employers and workers. However, in reality, factory management do not involve the workers to ensure effective OHS services, implying that they do not believe that the workers have responsibilities, rights or a role in OHS services. Further, the industry peak body (BGMEA) reported a need for improved government oversight and capacity to protect workers through provision of sufficient government OHS services suggesting that the industry was more responsive to buyers’ requirements than those of the government:

*“The government has insufficient capacity to monitor the working environment of the factories with only one hundred labour inspectors for over 5000 factories. Without a sufficient level of oversight by government, there is little incentive for employers to be accountable for ensuring the health of their workers. Instead, factory practices are driven by the demands of the buyers who are generally most interested in profit margins, and who if are not satisfied, will take the work contracts elsewhere.”*

## Discussion

Capacity is a key component of strengthening health systems, and data is a critical element in OHS management [21]. There is a significant human resource challenge for factory monitoring and supervision in the RMG industry in Bangladesh. Despite the significant increase in staffing levels in DIFE following the Rana Plaza incident, the shortage of properly qualified professional staff limits the effectiveness of the government’s efforts. There is also limited facility for targeted on-the-job training and mentoring, which is critical for strengthening capacity in ensuring a healthy and safe working environment. Moreover, the shortage of even basic monitoring equipment limits the inspectorate’s ability to carry out effective monitoring of the hazards and health and safety risks of workers in the RMG. The results of this research highlight some specific areas for building skills and capabilities.

It is no surprise that these findings imply that the OHS surveillance and national reporting system in Bangladesh is not functioning well. To make evidence-based policy, the government needs sufficient and accurate data [22]. This research identifies that the system for collecting and managing data is weak, and government staff are not well enough trained or equipped to collect, analyse or interpret data. These findings are consistent with other studies in low-income settings where the efficient use of limited resources to attain health goals is a challenge [23].

Finally, the findings of this research highlight the competing realities among the government, employers, and factory doctors in ensuring the health and safety of the workers. It is clear that the DIFE government officials lack power to enforce the law. The employers for their part insist that they do not have enough profit to invest in the health and safety of their workers. They argue that they are currently caught in a squeeze between overseas buyers who are unwilling to pay more for the garments and the cost of labour, and they worry that additional resource for OHS for their workers would reduce their profitability. The factory doctors meanwhile can see the health problems at an individual factory level and are concerned about long term health consequences for this workforce, but feel powerless to do anything about it. Their primary loyalty appears to be to the employer who pays their salary, not the workers.

The findings of this research have implications for how to improve the capacity of the DIFE. The government needs to recognize the challenges in ensuring the safety and health of the workers. As long as the DIFE is under-resourced and kept powerless, it is unable to ensure a better working environment for the female workers in the RMG. More collaboration and partnership with the development partners targeting individual capacity through training will enhance the capacity of the DIFE. The doctors of the factories understand the health problems that are developing among workers and hence they need to be made part of the government surveillance system in order to provide necessary data on occupational health and safety problems in this important sector.

### Suggested recommendations

The DIFE appears powerless to enforce the rules. Increasing its authority and improving penalty options, including direct enforcement action will empower them to ensure that suitable OHS services are provided. The situation described in our study suggests that significant changes in organizational culture, resourcing and strategy are required.

The lack of medical human resources with sufficient OHS expertise within relevant government agencies needs to be addressed as a priority. Strategies to appropriately fund, attract and train medical and health professionals to work under the Ministry of Labour and Employment, are urgently needed.

Regarding capacity building training, a solution offered by key informants is to establish an OHS training institute in the country. Such an endeavour could be developed in partnership between universities, government, and industry to ensure a suitable and available OHS workforce that contributes to more sustained OHS outcomes.

Finally, suggested solutions to surveillance and the national reporting system related barriers should focus on developing standard procedures and building capacity of the DIFE staff for collecting, storing, and reporting of OHS related problems of RMG workers so as to take more policy-oriented actions to protect this valuable workforce.

In order to better understand the organizational influence of health impacts relating to workplace conditions for the RMG predominantly female workforce, it would be useful to quantify the amount of time that women are working sub-optimally i.e. a phenomenon commonly referred to as ‘presenteeism’ or not at all (i.e. absenteeism due to work-place related injury) to provide stronger economic evidence of the costs to production of not ensuring adequate workplace health and safety protection. In other industries and settings this type of information has provided powerful evidence to persuade corporations that ensuring adequate workplace conditions and making workplaces attractive to employees has economic benefits [24].

### Limitation of the study

This study explored the situation in one district; the findings are therefore not representative of the whole country. However, the findings of this study provide practical observations on OHS system preparedness, highlighting the areas that need strengthening to ensure health and safety of the workers.

## Conclusion

This paper has identified a critical gap between needs and available resources in ensuring OHS services in Bangladesh. Changing patterns of employment have created new challenges, and employment in the RMG industry exposes workers to health hazards that may result in injuries, respiratory diseases and other ailments [5]. The government of Bangladesh is trying to improve the service of the department with their limited resources but despite some improvements in OHS service in the RMG industry, there are still significant challenges to be addressed. These include improving the capacity of DIFE to collect appropriate data, keep records and analyse them effectively, providing inspectors with enough skills and equipment, and giving inspectors sufficient power to enforce regulations.

This study will benefit national and international stakeholders by enabling them to understand the current status of the OHS service in the Bangladesh RMG sector, and to understand the level of progress and the continuing challenges in ensuring workers’ health. This includes global health dimensions, recognising that the low prices consumers pay for garments in HICs have consequences for the safety and long term health security of workers in this industry in LMICs.

## Additional file

Additional file 1: Guideline of Key Informant Interview. (DOCX 12 kb)

## Data Availability

The data are not publicly available due to restraint of information that could compromise research participant anonymity and privacy but are available from the corresponding author on reasonable request.

## Abbreviations

BGMEA: Bangladesh Garment Manufacturers and Exporters Association
DIFE: Department of Inspections for Factories and Establishments
GDP: Gross Domestic Product
ILO: International Labour Organization
OHS: Occupational Health and Safety
RMG: Ready-made Garment
WHO: World Health Organization
