# Supplementary material for: Exploring the system capacity to meet occupational health and safety needs: the case of the ready-made garment industry in Bangladesh
Source: BMC Health Serv Res. 2019 Jun 28;19:435. doi: 10.1186/s12913-019-4291-y (PMC6599266; doi:10.1186/s12913-019-4291-y)
Supplement: Supplementary file 1 — Guideline of Key Informant Interview. (DOCX 12 kb) [file 12913_2019_4291_MOESM1_ESM.docx]

**Guideline of Key Informant Interview**

Date: ____________________

Interviewer Name: _____________________

Respondent name: ______________________________

Age:

Occupation:

Religion:

Education:

**Information related to OHS system readiness work: purpose and rationale**

To establish rapport with respondents through questions those are relatively easy for them to answer:

To know respondents’ current situation of OHS, staff and their challenges as a basis for starting the conversation within the larger sphere of concern of this study, (which we call OHS system capacity) I could start the discussion by saying, ‘I’d like to start by asking can describe me the current capacity of DIFE to address the issues of OHS in Dhaka district in regard to RMG sector?

What are the current programs in Bangladesh to improve the working condition for women in the garment industry?

**Information related to OHS system related challenges**

What barriers and challenges do you have to establish a OHS system in Bangladesh to improved working conditions do you identify?

What does the government need to do to improve the working conditions in the garment industry in Bangladesh? (Probe: human resources, training need)

**Information related to monitoring, surveillance and reporting:**

How do you monitor the RMG factories?

How do you collect the information of health and safety-related problems of the garment workers who are working in the garment industry?

Do you have monitoring check-list? Can you describe content of the monitoring checklist?

What policy do you have for the health and safety of the workers of your factory?

What gender policy do you have for the women who are working in the garment industry?

What compensation policy do you have regarding the health and safety issues for your factory?

How frequently the government officials visit the factories?

What do they monitor when they come to visit your factory?

What initiatives have been taken in your factory to avoid incidents like the Rana Plaza collapse?

What is your opinion about the overall safety issues of the garment industry in Bangladesh?

What are your suggestions to improve the health and safety of the garment industry in Bangladesh?

When did you establish your garment factory?

How did you come into this business?

How many factories do you have?

How many workers are working in this factory?

How many of them are women?

Why do you recruit women for your factory?

How do you recruit these women?

What benefit do you provide to the women other than their salary?

What problems do you encounter when you recruit women for this industry?

What work-related health problems do the women have?

What health benefits do you provide for the women who are working in your factory?

What safety problems do female workers encounter at their workplace?

**Information related to suggestion challenges to establish OHS system:**

What needs to be done to improve the OHS system related situation for RMG sector in Bangladesh?

What type of training needed for the government staff for DIFE?
